# Zebrafish adult-derived hypothalamic neurospheres generate gonadotropin-releasing hormone (GnRH) neurons

**DOI:** 10.1242/bio.010447

**Published:** 2015-07-24

**Authors:** Christian Cortés-Campos, Joaquín Letelier, Ricardo Ceriani, Kathleen E. Whitlock

**Affiliations:** 1 Centro Interdisciplinario de Neurociencia de Valparaíso (CINV), Facultad de Ciencias, Universidad de Valparaíso, Pasaje Harrington 269, Valparaíso 2340000, Chile; 2 Whitehead Institute for Biomedical Research (WIBR), 9 Cambridge Center, Cambridge, MA 02142, USA; 3 Centro Andaluz de Biología del Desarrollo, Universidad Pablo de Olavide, Carretera de Utera km 1, Sevilla 41013, España

**Keywords:** Kallmann syndrome, GnRH receptors, Testosterone

## Abstract

Gonadotropin-releasing hormone (GnRH) is a hypothalamic decapeptide essential for fertility in vertebrates. Human male patients lacking GnRH and treated with hormone therapy can remain fertile after cessation of treatment suggesting that new GnRH neurons can be generated during adult life. We used zebrafish to investigate the neurogenic potential of the adult hypothalamus. Previously we have characterized the development of GnRH cells in the zebrafish linking genetic pathways to the differentiation of neuromodulatory and endocrine GnRH cells in specific regions of the brain. Here, we developed a new method to obtain neural progenitors from the adult hypothalamus *in vitro.* Using this system, we show that neurospheres derived from the adult hypothalamus can be maintained in culture and subsequently differentiate glia and neurons. Importantly, the adult derived progenitors differentiate into neurons containing GnRH and the number of cells is increased through exposure to either testosterone or GnRH, hormones used in therapeutic treatment in humans. Finally, we show *in vivo* that a neurogenic niche in the hypothalamus contains GnRH positive neurons. Thus, we demonstrated for the first time that neurospheres can be derived from the hypothalamus of the adult zebrafish and that these neural progenitors are capable of producing GnRH containing neurons.

## INTRODUCTION

The hypothalamus integrates information essential for the control of homeostasis including blood pressure, appetite, social behaviors, and reproduction. Multiple nuclei, including the neuroendocrine magnocellular and parvocellular nuclei, regulate complex processes through direct synaptic contacts as well as release into tissues and portal systems (Garcia-Segura, 2009; Machluf et al., 2011). The gonadotropin-releasing hormone (GnRH) cells of the parvocellular nucleus in mammals regulate puberty and fertility through pulsatile hormonal release via projections in the median eminence into the hypophyseal-portal-vasculature system, resulting in the release of the target hormones from the adenohypophysis. Although structurally different from mammals, the brain of teleost fish contain all the hypothalamic cell types (Machluf et al., 2011) including GnRH cells localized to the parvocellular nucleus (Gomes et al., 2013).

In humans, the congenital failure in the function of the GnRH neuroendocrine system results in reproductive disorders termed hypogonadotropic hypogonadism (HH), and these patients can also show a wide variety of non-reproductive phenotypes. Within the HH phenotype there exists a congenital GnRH deficiency with associated anosmia called Kallmann syndrome, which is now known to be a heterogeneous disease (Balasubramanian et al., 2010). Subsequent analysis of human patients have shown that HH results from mutations falling into two basic categories: those that affect peptides and/or ligands (*GnRH*, *kisspeptin*, *prokineticin2*) and those that affect the patterning and early development of the brain (*fgfr1*, *fgf8*, *anosmin1*, *CHD7*). In the case of mutations in the ligand receptor pairs of *kisspeptin* and *GnRH*, the defects are restricted to the loss of the pulsatile GnRH secretion necessary for the onset and maintenance of puberty. In contrast, mutations affecting brain patterning affect not only GnRH cell development but also cause a variety of associated phenotypes: cleft lip, cleft palate, high arched palate and other midline defects (reviewed in Silveira et al., 2010).

Males suffering from infertility associated with idiopathic HH (IHH) undergoing hormone therapy using testosterone, GnRH, or both, can show a reversal of IHH accompanied by a restoration of pulsatile GnRH release. Unexpectedly, these patients can continue to show pulsatile GnRH after removal of hormone treatment (Raivio et al., 2007) suggesting that the hypothalamus can recover GnRH function in adult humans.

The ability to generate new GnRH cells in the adult brain would require a quiescent progenitor population in the hypothalamus. It is widely accepted that adult neurogenesis occurs in the subventricular zone of the lateral ventricles and the subgranular zone of the hippocampus. More recent *in vivo* studies support the hypothalamus as a source of neurogenic and gliogenic precursors (Pérez-Martín et al., 2010; Sousa-Ferreira et al., 2014, 2011; Xu et al., 2005). The discovery of proliferating and neural stem cell (NSC) populations in the hypothalamus have been linked to the maintenance of body weight and energy expenditure (Bolborea and Dale, 2013). The observations that the vertebrate brain has the ability to generate new neurons have led us to examine genesis of GnRH cells in the adult zebrafish. To date no convincing studies have shown GnRH positive cells in the preoptic area (POA) nuclei of the adult zebrafish hypothalamus, though it has been suggested that these cells migrate to this region (Abraham et al., 2009) and that the hypothalamus in fact does not contain GnRH positive cell bodies. Here we show that GnRH cells can be detected by immunocytochemistry in the POA of adult zebrafish, that neurospheres can be isolated from the adult hypothalamus and differentiate into GnRH cells, that the number of GnRH cells increases in a dose dependent manner following hormone exposure (testosterone/GnRH), and that there is a potential neurogenic niche for GnRH cells in the hypothalamus of the adult brain. These data support a model where new, centrally derived GnRH cells can be generated in response to hormone treatment in IHH patients.

## RESULTS

Detection of endocrine GnRH cells in the hypothalamus of zebrafish using antibodies has been famously inconsistent. A potential explanation for the difficulties of GnRH immunolocalization in the POA is that the expression of GnRH peptide in cell bodies is extremely variable as has been suggested in Medaka (Karigo et al., 2012). We have discovered, through analysis of the reproductive state, that GnRH immunoreactivity in the hypothalamus varies depending on mating behavior and light cycle, most likely due to the cyclical nature of peptide release. Thus, for this study we worked only with males and only with animals selected to successfully fertilize eggs (see Materials and Methods; mating training). With careful attention to the light cycle and reproductive history of the fish, we were able to consistently visualize GnRH immunoreactive cells in the hypothalamus.

**Fig. 1.**
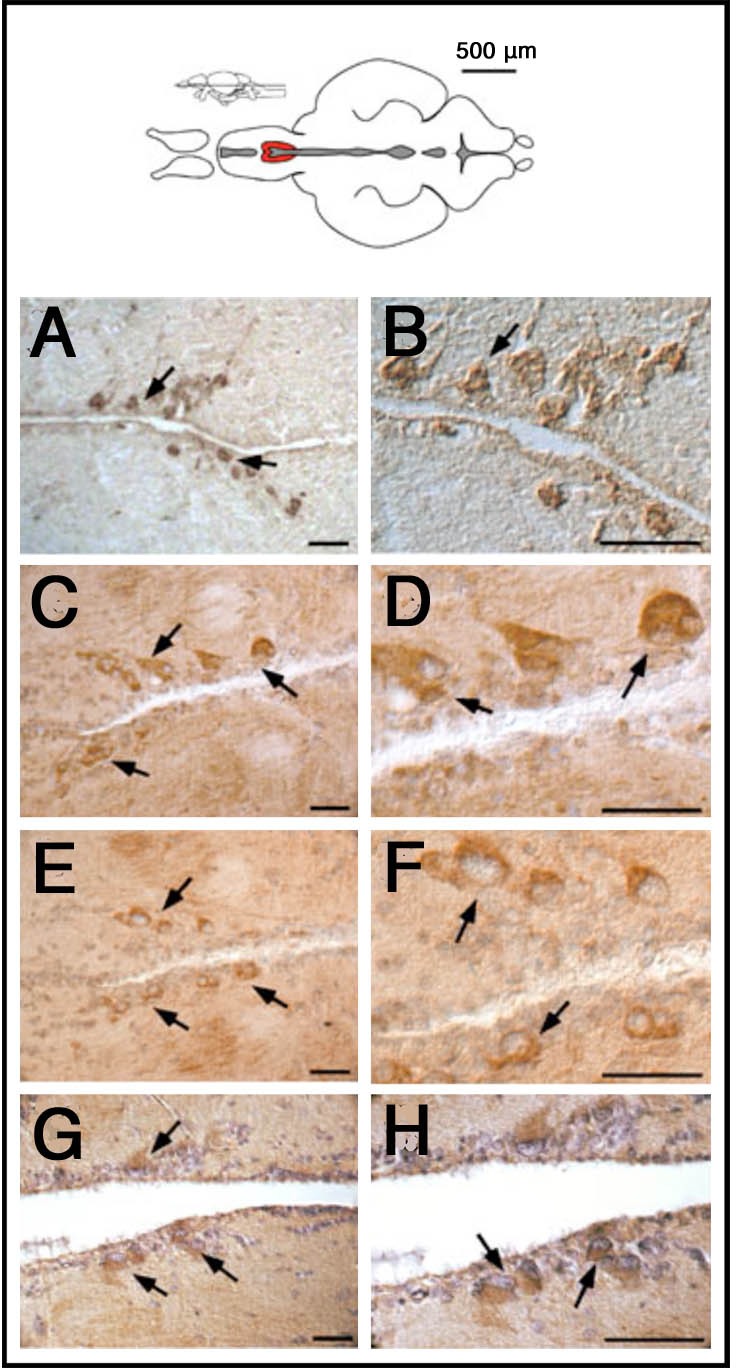
**GnRH antibodies recognize cells in the anterior preoptic region of the adult brain.** Brain sections (A,B, cryostat sections; C-G, paraffin sections) of different animals immuno-stained with different antibodies against GnRH: (A,B) anti-GnRH (LRH13); (C,D) anti-GnRH (Hu11B); (E,F) anti-mGnRH (Sigma-Aldrich); (G,H) anti-sGnRH (BB8). All antibodies show immunoreactivity in neurons (arrows) localized in the hypothalamic POA (diagram, red). Scale bars: 50 μm.

To analyze the expression of GnRH in the hypothalamus of the adult zebrafish we used different antibodies known to recognize hypothalamic GnRH in mammals and fish: anti-GnRH (LRH13); anti-GnRH (Hu11B); anti-mGnRH; anti-sGnRH (BB8). When used on sections of adult brains, these antibodies recognize a small population of cells in the POA (Fig. 1). This population of GnRH containing cells shows a consistent pattern of immuno-labeling in both cryostat and paraffin embedded sectioned tissue. This pattern does not overlap with that of either *GnRH2* or *GnRH3* gene expression (Gopinath et al., 2004) or that of transgenic reporter line expression (GnRH3:GFP; Abraham et al., 2008; Zhao et al., 2013), and therefore could correspond to a hypothalamic GnRH isoform in zebrafish. Thus we have confirmed the presence of GnRH in the parvocellular nucleus of the POA.

**Fig. 2.**
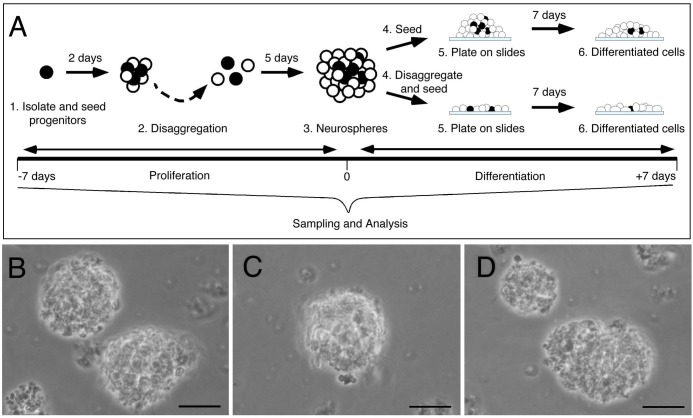
**Neurospheres can be generated from the hypothalamus of adult zebrafish.** (A) Neural progenitors were disaggregated (black cells), plated, and maintained in proliferation media for 7 day (A1 to A3 neurospheres, −7 to 0 days). Cells were then seeded (A4 to A6, upper panel) on coated chamber wells and changed to differentiation media for 7 days (0 to +7 days). Alternatively, neurospheres (A3) were disaggregated and seeded (A4 to A6, bottom panel) on coated chamber wells and changed to differentiation media for 7 days with or without hormonal treatment (0 to +7 days). (B-D) 7 days *in vitro* bright field images of neurospheres (A3). Scale bar 30 μm.

To explore the possibility of GnRH neurogenesis in the adult zebrafish we developed a method to obtain neural progenitors from the brain of adult zebrafish. We dissected the region of the hypothalamus from fish aged one to two years and dissociated the cells mechanically to select individual cells for seeding (Fig. 2A1, black cells: progenitors). The resulting primary neurospheres, formed over two days, were mechanically disaggregated to individual cells (Fig. 2A2, white cells: progenitors slightly differentiated) and then seeded in cell culture chambers for 5 days resulting in undifferentiated secondary neurospheres (Fig. 2A3,B-D).

**Fig. 3.**
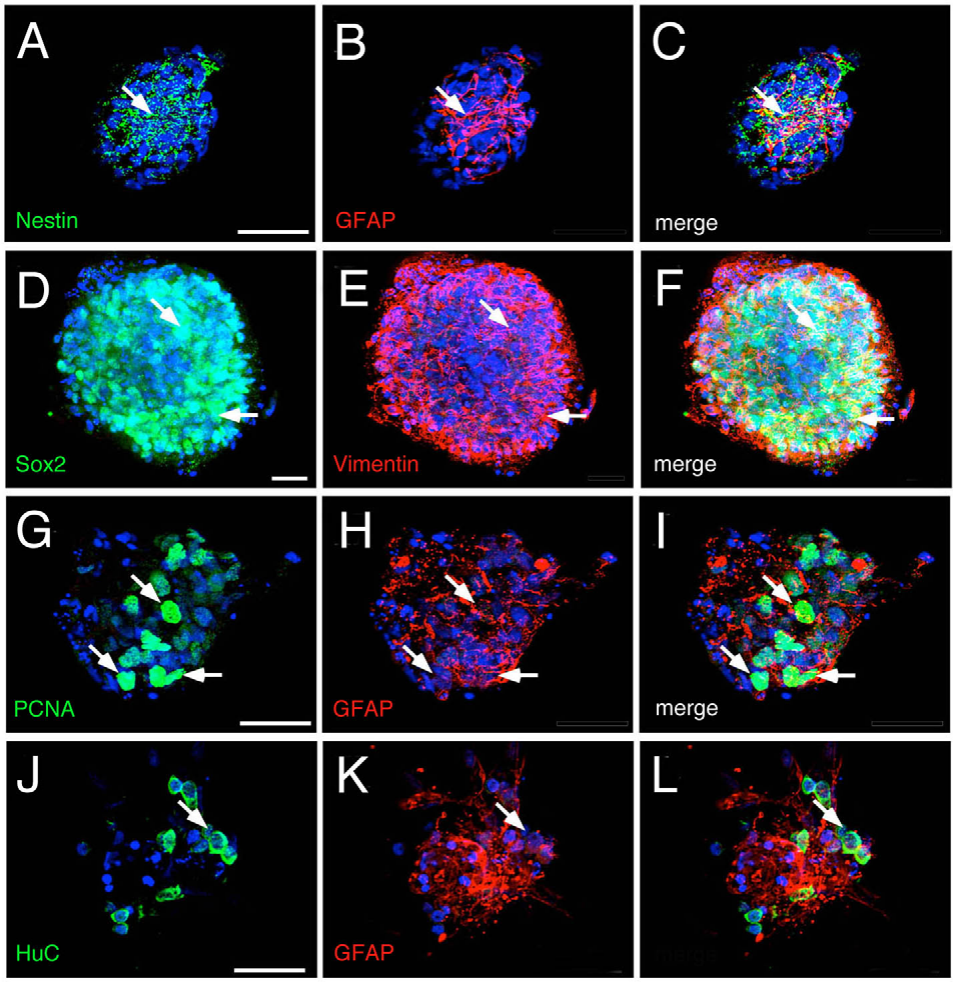
**Hypothalamic neurospheres express neural progenitor markers.** Undifferentiated 7 days *in vitro* neurospheres. Cells expressing nestin and GFAP are located principally at the center of the neurospheres, (A-C, arrows). Sox2 and vimentin, markers of undifferentiated neural progenitors, are expressed in the neurospheres (D-F, arrows). Neurospheres are highly proliferative, expressing PCNA (green) in many of the GFAP positive (red) cells (G-I, arrows). Undifferentiated neurospheres reactive to GFAP show a reduced number of neurons (positive for HUC) (J-L, arrows). Scale bar 25 μm; *n*=3 plates derived from different cultures.

To characterize the cell types present in the undifferentiated secondary neurospheres (Fig. 2A, time=0), they were seeded (see Materials and Methods) on a L-poly-l-lysine/laminin coated substrate for 6 h and fixed at room temperature for 30 min (Fig. 2A4-5, upper panel). Cultured cells were then processed for standard markers of stem cells: nestin, GFAP, Sox2 and vimentin (García-Verdugo et al., 1998; Remboutsika et al., 2011; Scheffler et al., 2005). Nestin, a type VI intermediate filament (IF) protein is expressed in undifferentiated precursors (Fig. 3A,C, green, arrows). GFAP, a type II IF protein with various splice products where the GFAP-delta form is associated with neurogenic astrocytes (Kamphuis et al., 2012) is expressed in undifferentiated neurospheres (Fig. 3B,C,H,I,K,L, red), as is the type II IF protein vimentin (Fig. 3E,F, red). These neurospheres show high levels of Sox2 expression (Fig. 3D,F, green, arrows) and PCNA (proliferating cell nuclear antigen) expression (Fig. 3G,I, green, arrows), supporting an undifferentiated state. HuC, a developing neural specific RNA-binding protein, (Fig. 3J,L green, arrows) is expressed in very few cells (0.22±0.05 HuC+/total cells). These undifferentiated secondary neurospheres do not express GnRH (supplementary material Fig. S1).

**Fig. 4.**
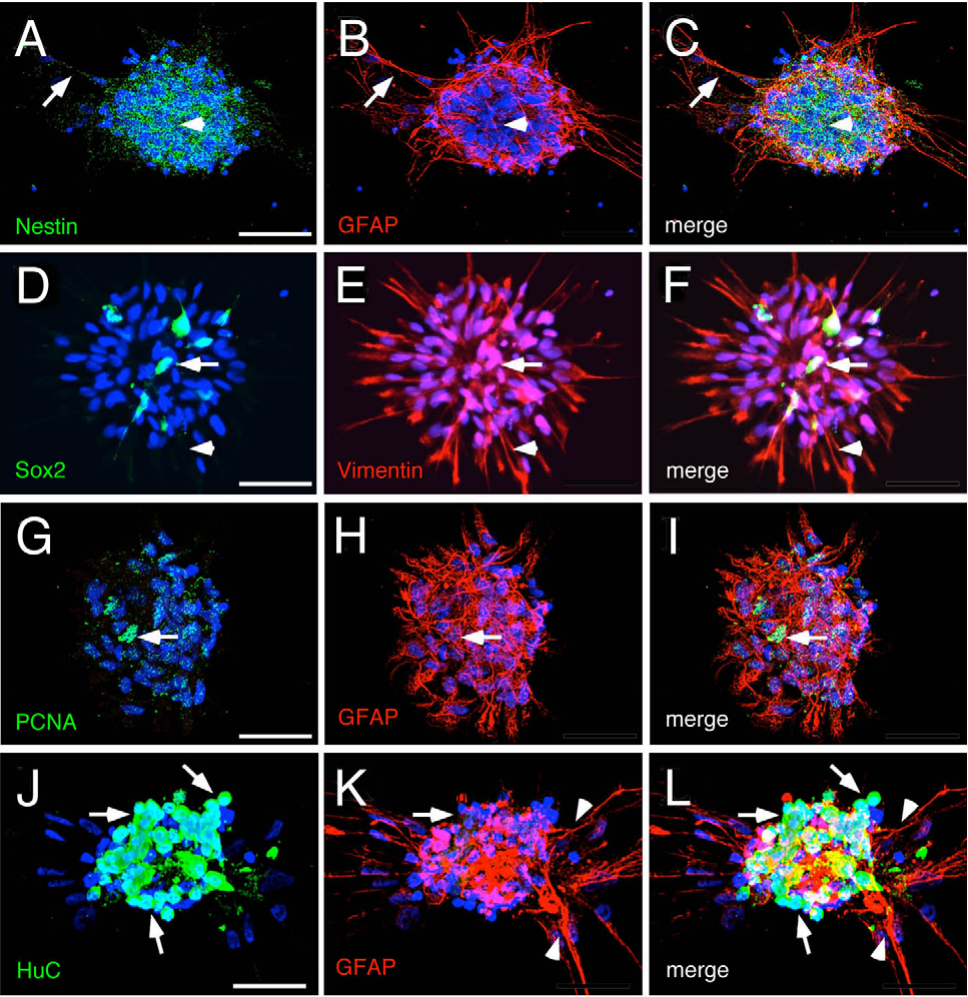
**Hypothalamic progenitors generate glial and neuronal cells.** (A-F) Neurospheres differentiated after 7 days *in vitro*. (A-C) Differentiated cells surround the core of cells positive for nestin (arrow head) and extend cellular projections that are reactive to GFAP (arrows). (D-F) The reduced number of Sox2 positive cells (arrows) and the high number of cell processes reactive to Vimentin (arrow heads) suggests that progenitors have differentiated into glial cells. (G-L) Neurospheres reactive to GFAP show (G-I) a reduced number of cells positive for PCNA (arrows), (J-L) high number of neurons (arrows) and differentiated glial cells (arrow heads). Scale bar 25 μm, *n*=3 plates derived from different cell cultures.

In order to determine whether the neurospheres could generate different cell types, they were cultured in differentiation media (see Materials and Methods) on a L-poly-l-lysine/laminin coated substrate (Fig. 2A4-6, upper panel). Cells were then fixed after 7 days and processed for different markers. The expression pattern of the IF proteins nestin (Fig. 4A,C, green), GFAP (Fig. 4B,C, red) and vimentin (Fig. 4E,F red, arrowhead) changed as glia differentiated and began to elongate their process (Fig. 4A-C arrows, D-F, arrowheads). Concurrently, the number of cells expressing Sox2 (Fig. 4D,F, green, arrow) and PCNA (Fig. 4G,I, green, arrow) decreased from 0.87±0.09 to 0.06±0.05 (Sox2+/total cells) and from 0.32±0.09 to 0.07±0.04 (PCNA+/total cells), respectively. In agreement with the differentiation of newborn neurons, the number of cells expressing HuC (Fig. 4J,L, green, arrows) increased (0.61±0.04 HuC+/total cells). The change in morphology and in expression of markers correlated with the differentiation of neurons and glia was observed in all differentiated cultures (*n*=3), and is consistent with media-induced differentiation observed in other studies (Louis and Reynolds, 2005).

**Fig. 5.**
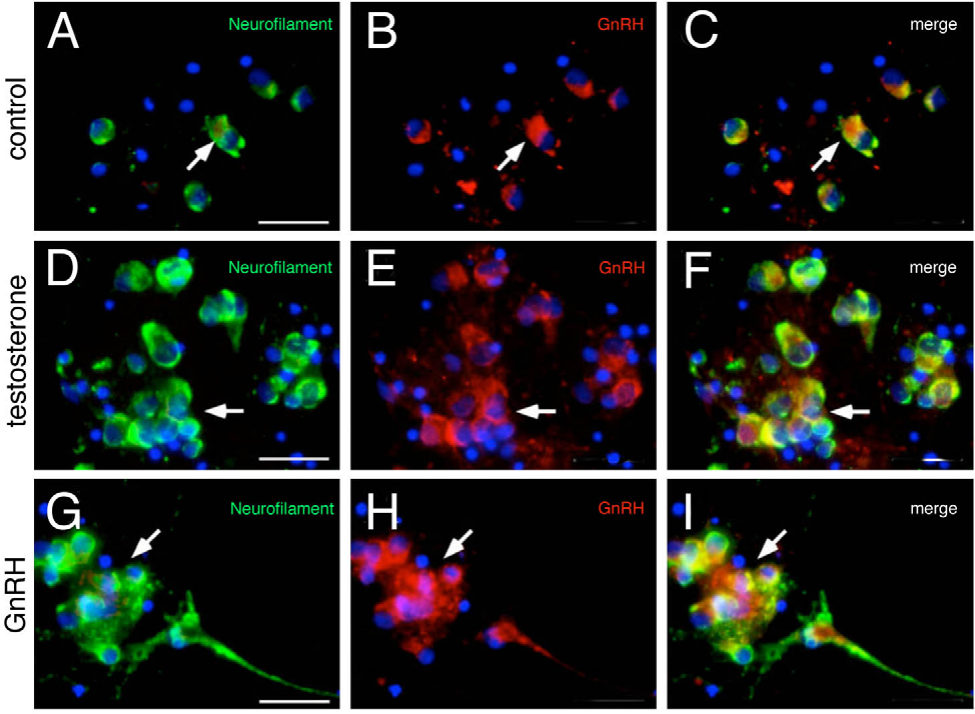
**Hypothalamic progenitors can differentiate GnRH neurons.** (A-C) Neurospheres differentiated for 7 days *in vitro* without hormonal stimulus. Neurospheres generate GnRH neurons (arrows). (D-F) Differentiated 7 days *in vitro* neurospheres, stimulated for 5 days with 10 μM testosterone. Testosterone increases the number of GnRH neurons in NSC cultures (arrows). (G-I) Differentiated 7 days *in vitro* neurospheres, stimulated for 5 days with 10 nM GnRH increases the number of GnRH neurons in NSC culture (arrows). Scale bar 25 μm.

Once the differentiation of neurons and glia was confirmed, subsequent experiments were carried out to determine whether the differentiated neurospheres were immunoreactive for GnRH. Cells from disaggregated neurospheres differentiated 7 days *in vitro* without hormonal stimulus (Fig. 2A3-6, bottom panel), were fixed and processed for GnRH immunoreactivity using the BB8 antibody (Kah et al., 1986). Strikingly, differentiated progenitor cultures contained GnRH positive cells (Fig. 5B,C, red, arrow) that were also positive for neurofilament (Fig. 5A,C, green, arrow) confirming their identity as GnRH positive neurons.

**Fig. 6.**
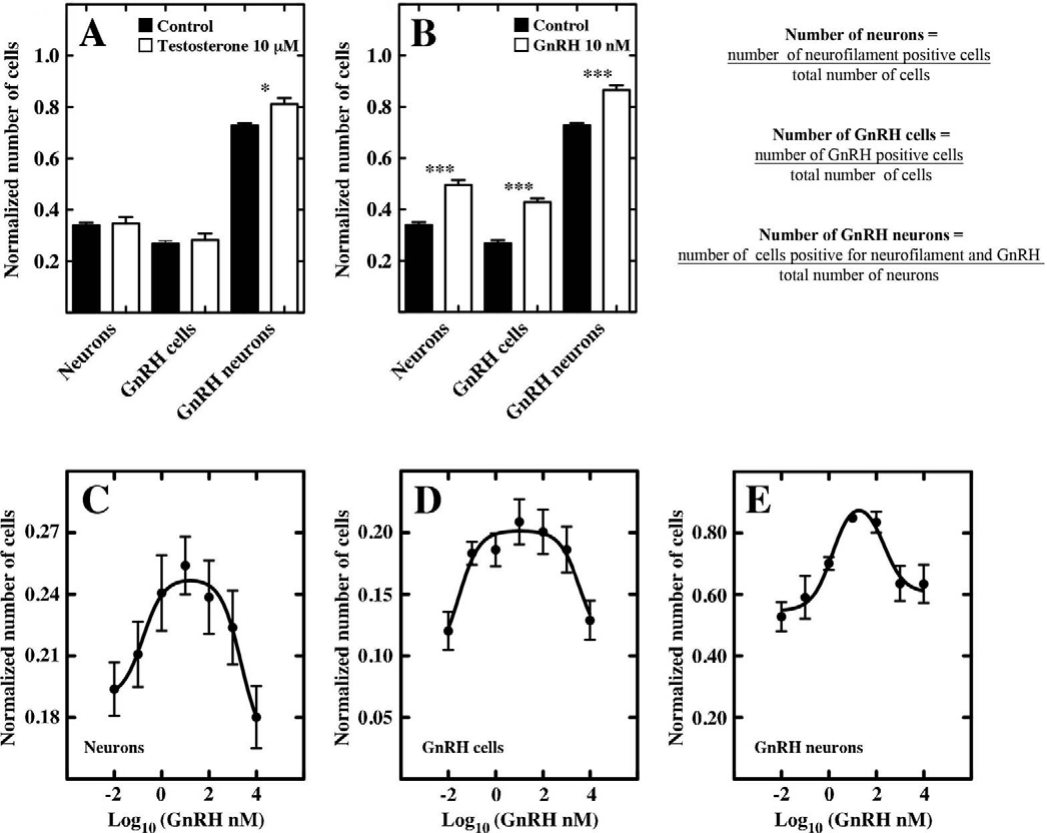
**Hormonal treatment increases the number of GnRH neurons.** (A) Neurospheres differentiated 7 days *in vitro*, and stimulated for 5 days with 10 μM testosterone showed an increase in the number of GnRH neurons. (B) Differentiated 7 days *in vitro* neurospheres stimulated for 5 days with 10 nM GnRH increase the number of neurons, GnRH cells and GnRH neurons. (C-E) Dose response plot of differentiated 7 days *in vitro* neurospheres, stimulated for 5 days with GnRH. All parameters analyzed show a bell-shaped dose response curve, with the maximum response at 10 nM. *P<0.05, ***P<0.0001. A,B: *n*=12 plates derived from 3 different cell cultures; C-E: *n*=10 plates derived from 4 different cell cultures. Error bars represent s.e.m.

In order to examine *in vitro* the potential effects of hormones used in the treatment of Kallmann syndrome patients (Raivio et al., 2007), we applied testosterone and GnRH to the neurosphere cultures to determine whether there was an increase in GnRH-positive neurons. For the hormone exposure experiment, GnRH3 was used, as it is currently the proposed potential endocrine form of GnRH in zebrafish. First, using values from the literature (Cheung and Wong, 2008) we exposed cells to control (vehicle) or 10 nM GnRH during the differentiation phase (Fig. 2A3-6, bottom panel) by supplementation every 2 days (days 3, 5, 7). In separate experiments cells were differentiated in the presence of 10 µM testosterone (T) (Mouriec et al., 2009) using the same protocol. The resulting differentiated cells showed an increase in the GnRH-positive neurons following exposure to either testosterone (Fig. 5D-E, arrows) or GnRH (Fig. 5G-I, arrows) relative to the control (Fig. 5A-C, arrows). The increase caused by testosterone was small but significant (Fig. 6A; *P*<0.05, *n*=12); in contrast, cells treated with GnRH showed a significant increase in the number of neurons and GnRH positive neurons (Fig. 6B; *P*<0.0001 for all neurons, *n*=12). Next, to determine whether there was a dose response in the number of GnRH cells generated, neurospheres were exposed to varying concentrations of GnRH during the differentiation phase and analyzed (Fig. 6C-E; *n*=10). The maximal effect was obtained using a final concentration of 10-20 nM (Fig. 6E), in agreement with values reported in the literature (Cheung and Wong, 2008).

**Fig. 7.**
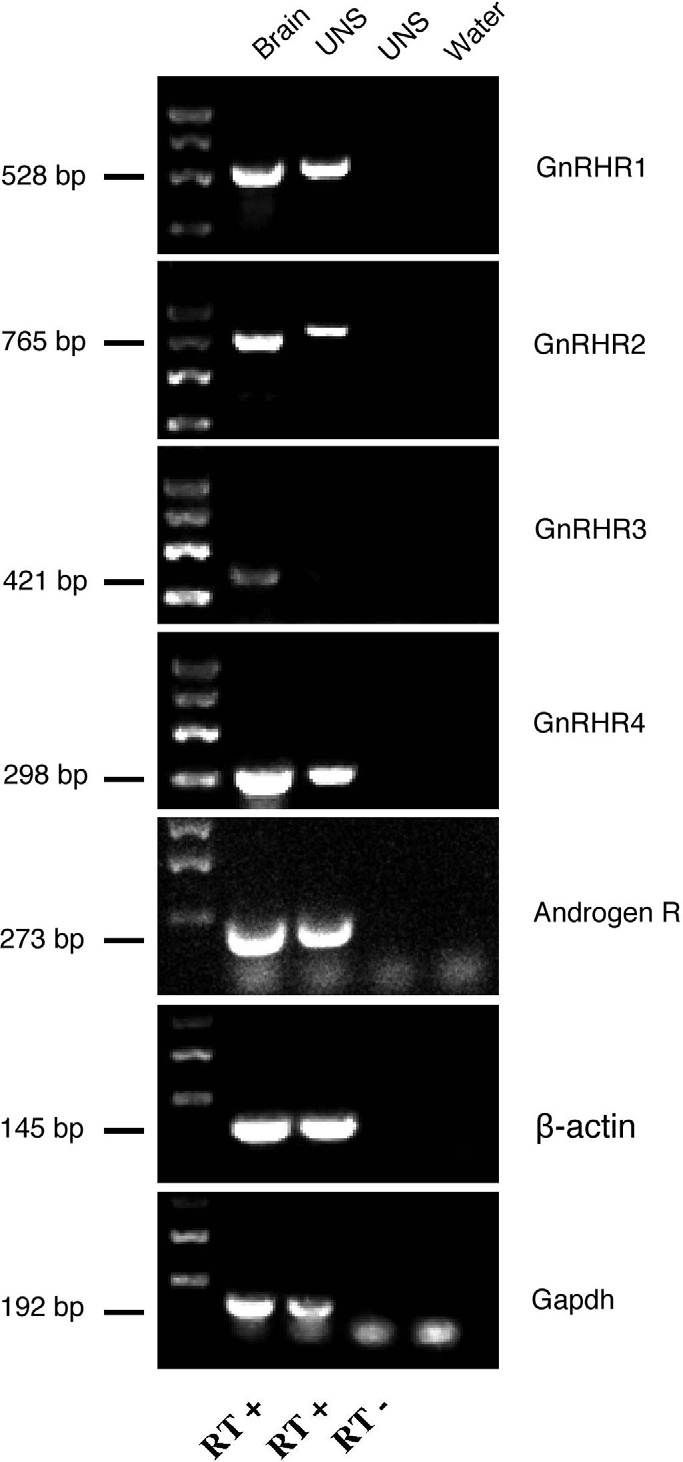
**GnRH receptors are expressed in undifferentiated neurospheres.** RT-PCR analyses of the four *zfGnRHR*s and *zfAR*. All *zfGnRHR*s are expressed in the brain of adult zebrafish. *zfGnRHR1*, *zfGnRHR2* and *zfGnRHR4* are expressed in undifferentiated neurospheres (7 days *in vitro*). UNS, neurospheres; RT+, positive reverse-transcription; RT−, negative reverse transcription. *β-actin* and *Gapdh*: house-keeping genes.

Because the neurospheres responded to testosterone and GnRH we examined whether the undifferentiated neurospheres (Fig. 2A4) express one or more of the androgen and/or GnRH receptors. We amplified *zfGnRHR1*–*zfGnRHR4* from undifferentiated neurospheres and whole brain of adult fish (Fig. 7) using previously published primers (Tello et al., 2008). The products resulting from the amplifications were sequenced and their identity confirmed. In agreement with the literature, all four *zfGnRHR*s were expressed in the whole brain tissue with *zfGnRHR3* being expressed at very low levels (Fig. 7, Brain). In contrast, only *zfGnRHR1*, -*2*, and -*4* were expressed in undifferentiated neurospheres (Fig. 7, UNS). The absence of *zfGnRHR3* expression *in vitro* is consistent with previous reports showing *zfGnRHR3* is highly expressed in the eye but is almost absent in the brain (Tello et al., 2008).

**Fig. 8.**
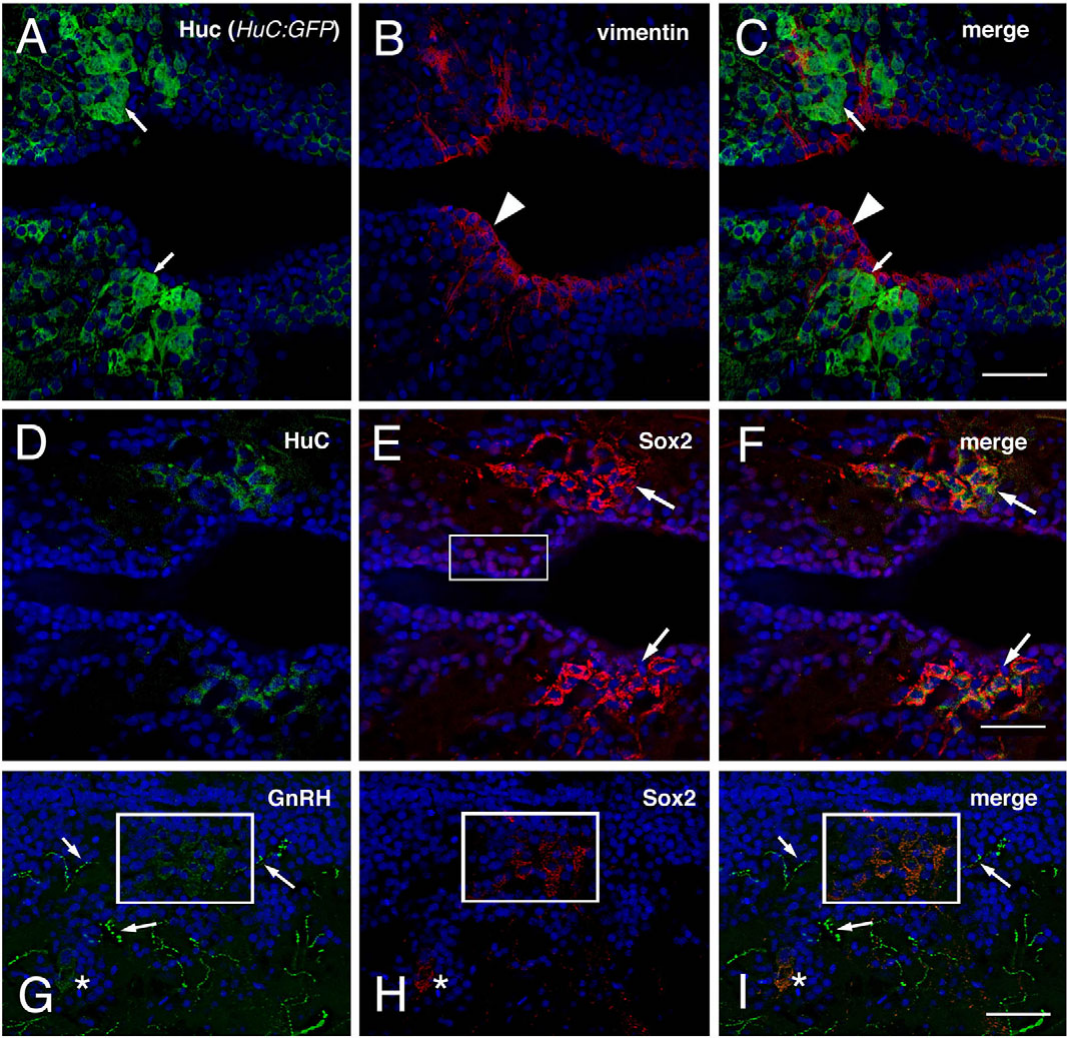
**The hypothalamus of the adult zebrafish contains newly differentiated GnRH cells.** Horizontal sections showing the anterior region of the parvocellular preoptic nucleus (PPa), with DAPI labeling (blue). (A-C) HuC labeling in a *HuC:GFP* background (green, arrows) and vimentin labeling (red, arrowhead) do not co-localize. The vimentin positive cells line the edges of the ventricles. (D-F) HuC (green) and Sox2 labeling (red, arrows) co-localizes in some cells (arrows) located closer to the border of the ventricle (arrows). (G-I) Section showing GnRH cell body labeling (green, box, asterisk) and labeling of processes (green, arrows), Sox2 labeling (red, box, asterisk) in cells located several cell diameters from the border of the ventricle (arrows) co-localizes with the GnRH labeling (merge). All Images: anterior is to the left. Scale bar=30 μm.

To investigate whether the hypothalamus of the intact adult zebrafish contains potential GnRH progenitors we used adult male zebrafish that had been selected via the mating training protocol and sacrificed them to obtain brains for sectioning. Cryostat sections were analyzed using antibodies against GnRH, Sox2, and HuC. Cells positive for HuC labeling (Fig. 8A, arrows) were localized in the anterior region of the parvocellular preoptic nucleus (PPa) and these cells were distinct from the vimentin positive glia (tanycytes) lining the ventricle (Fig. 8B,C, red, arrowheads). Immuno-localization of the progenitor marker Sox2 showed nuclear label in the cells lining the ventricle (Fig. 8E, boxed area for example) and cytoplasmic localization in cells located several cell diameters from the ventricle (Fig. 8E,F, arrows). A subset of the Sox2 positive cells co-localized with HuC positive cells (Fig. 8F) a marker for newly generated neurons in agreement the role of Sox2 as a neural progenitor. While cells lying adjacent to the ventricle had nuclear localization Sox2, cells lying more distant, such as HuC positive cells show a cytoplasmic expression of Sox2. Immuno-labeling for GnRH localized cell bodies (Fig. 8G, green, box, asterisk) and processes (Fig. 8G, green, arrows). Strikingly, a small population of cells positive for GnRH also immuno-labeled with Sox2 (Fig. 8H,I, boxed area, asterisk). Like HuC, GnRH co-localizes with cytoplasmic expression of Sox2 in cells located several cell diameters from the ventricular surface (Fig. 8A,D,G). Thus GnRH positive neurons are located in a neurogenic niche in the PPa of the hypothalamus in the brain of the adult zebrafish.

## DISCUSSION

Here we have shown that the hypothalamus of the adult zebrafish contains GnRH neurons, has the ability to generate neurospheres that can differentiate into new GnRH neurons, the number of GnRH positive neurons can be increased by hormone treatment, and that the intact animal can potentially generate new GnRH cells. These data support a model where the hypothalamus has the ability to generate GnRH cells in the adult animal, an observation having ramifications for human health, because of the potential to identify GnRH progenitors in adults.

### Neurogenesis

With the exception of the olfactory sensory neurons, which regenerate throughout life, it was believed for many years that neurogenesis did not occur in the central nervous system of vertebrates. With the striking discoveries of the neurogenic capacity in the song circuitry of birds (Alvarez-Buylla et al., 1988) and the chain migration of SVZ generated neurons in mammals (Lois and Alvarez-Buylla, 1993; Luskin, 1998) our thinking changed radically in regard to the regenerative capacities of the adult central nervous system (CNS). It has recently become apparent that the hypothalamus has a neurogenic potential in the adult animal, where NSC populations have been identified in both mouse and rat (Sousa-Ferreira et al., 2014). Fish differ from mammals in that they generate neurons throughout life as part of their indeterminate growth and they can recover from injuries to their CNS (Kizil et al., 2012). Zebrafish, like mammals have distinct proliferation zones in the adult brain, including the preoptic area of the ventral hypothalamus which includes the anterior parvocellular preoptic nucleus (Grandel et al., 2006). The neurospheres generated here were produced using cells from this region because it contains the GnRH cells in adult zebrafish as we have shown for the first time in this study, and because it was previously reported to have neurogenic potential (Adolf et al., 2006; Grandel et al., 2006). Here we showed that the neurospheres derived from the hypothalamus of the adult zebrafish contain GnRH progenitors and *in vivo* analyses support the presence of new GnRH cells generated in the adult brain similar to a recent report in the ring dove (Cheng, 2013; Cheng et al., 2011).

### Hormone induced proliferation

The response of the neurospheres to GnRH and testosterone was distinct with GnRH causing both an increase in neurons and an increase in the number of GnRH cells, thus an overall proliferation effect. In contrast testosterone causes only a small but significant increase in GnRH neurons. Our results are consistent with a reported biphasic effect of GnRH agonists on growth where GnRH agonists at high doses (1 µM) inhibit cell proliferation *in vitro*, and cells treated with agonists at low dose (10 nM) show a significant increase in proliferation (Cheung and Wong, 2008). Interestingly this GnRH induced increase in proliferation may be correlated with a recent finding showing that ectopic administration of GnRH via the third ventricle of old mice can prevent ageing-impaired neurogenesis predominantly in the hypothalamus and hippocampus (Zhang et al., 2013).

### GnRH receptors

The dramatic response of the cells to GnRH suggested a potential GnRH receptor (GnRHR) interaction at the level of the undifferentiated neurospheres. In zebrafish there are four *GnRH-R* genes (Tello et al., 2008) and they are all expressed in the brain as well as in non-neuronal tissues throughout the body. Here we have shown that three of the four reported GnRHRs, GnRHR1, GnRHR2, GnRHR4, as well as the androgen receptor (Gorelick et al., 2008) are expressed in the neurospheres generated from the adult hypothalamus. The GnRHRs are notoriously promiscuous where a given ligand (GnRH1, GnRH2, GnRH3) can bind several GnRHRs. GnRH1 stimulates zfGnRHR2 and zfGnRH4 but not zfGnRHR1 or zfGnRHR3 in the physiological range (Tello et al., 2008). Additionally, zfGnRHR1 and zfGnRHR3 show a higher affinity for the GnRH2 peptide, while zfGnRHR2 and zfGnRHR4 respond equivalently to both GnRH2 and GnRH3 ligands. Thus the expression of *zfGnRHR2* and *zfGnRHR4* in neurospheres is consistent with the known response characteristics of the endocrine GnRH ligand in zebrafish. In addition to the GnRH response, neurospheres also showed a response to testosterone albeit reduced relative to the GnRH response. In agreement with the literature (Gorelick et al., 2008) we found that brain tissue expressed androgen receptor (AR) and confirmed that ARs are expressed in neurospheres (Fig. 7). Exposure to testosterone has been reported to increase GnRHR mRNA expression in rats while leaving GnRH mRNA unaffected (Botté et al., 1999), but to also repress GnRH gene expression through direct interaction with the GnRH promoter (Brayman et al., 2012). Thus the effects of testosterone on differentiation may reflect the complex regulatory roles played by testosterone in the brain.

### Hypothalamic GnRH in zebrafish

The GnRH decapeptide is a highly conserved protein having both neuromodulatory and endocrine functions in vertebrates. The neuromodulatory GnRH cells are located in the terminal nerve, ventral telencephalon and synencephalon (Whitlock, 2005). In contrast, the endocrine GnRH cells responsible for the initiation of puberty and control of reproduction are located in the POA of the hypothalamus. Clinical data from infertility treatments showing a reversion of phenotype (restoration of GnRH pulsatility in the adult) suggest that there may be a GnRH progenitor population in the adult. Because it is highly unlikely that new GnRH cells would migrate in the adult from the olfactory epithelium, through the cribiform plate and to the hypothalamus, the most plausible explanation is that a GnRH progenitor population exists within the CNS.

In the literature, the immunocytochemical localization of GnRH in the hypothalamus of the adult zebrafish has been inconsistent, with varied expression patterns reported including the suggestion that the zebrafish have no GnRH positive cell bodies in the POA. In addition to immunocytochemical studies, reports using GFP reporter lines to visualize GnRH cells *in vivo* (Torgersen et al., 2002; Abraham et al., 2009, 2008; Ramakrishnan et al., 2010; Zhao et al., 2013) have also been inconsistent and do not agree with endogenous gene expression data (Gopinath et al., 2004; Steven et al., 2003). Using three different antibodies that detect GnRH, we have been able to unequivocally localize GnRH containing cell bodies in the parvocellular nucleus of the adult hypothalamus. A key factor is to consider the time of day at which the animal is sacrificed as well as its reproductive state. Here we have shown that by selecting for fertile fish, those that consistently lay high quality eggs (hence reflecting the quality of both the oocyte and sperm), and sacrificing the animal at a specific time of day, we can obtain highly reproducible GnRH labeling in the adult hypothalamus.

### New GnRH cells in the adult brain

Our *in vivo* analysis has identified a potential neurogenic niche in the PPa of the hypothalamus as reflected by the localization of Sox2, a transcription factor critical for the differentiation of pluripotent stem cells to neural progenitors as well as for maintaining neural progenitor stem cells (Zhang and Cui, 2014), with differentiating (HuC+) neurons and GnRH neurons. Recently it has been shown that adult mouse hypothalamus contains NSC populations that can generate new neurons in response to metabolic signals involved in the maintenance of body weight (Bolborea and Dale, 2013). A potential neural progenitor found in the ventral regions of the ventricle wall, tanycytes (a specialized type of glia), are interspersed with ependymal cells, another cell type known to have NSC properties (Carlen et al., 2009; Robins et al., 2013). A variety of hormones and peptides including GnRH (Pestarino et al., 1998) have been localized to tanycytes. GnRH has been shown to associate with beta1-tanycytes a relationship that is proposed to regulate the pulsatile release of GnRH from the terminals of the GnRH neurons (Rodríguez et al., 2005). Fish, while maintaining the NSC niches in the adult brain, differ from mammals in that they lack an ependymal layer separating the subventricular zone from the lumen of the ventricle, (Grandel and Brand, 2013; Lindsey et al., 2012). Yet, fish have tanycytes that are considered to be a subtype of radial glia, and recently a fish cell line has been shown to generate cells with tanycyte characteristics (Wen et al., 2010). We have shown that cells positive for vimentin, a marker for tanycytes, are located in the PPa in the adult zebrafish in close proximity to the neurogenic niche containing HuC positive cells and GnRH neurons. Furthermore this region shows active cell division as evidence by BrdU labeling (R.C., Ana Carolina Abbott and K.E.W., unpublished). Further *in vivo* studies in control and treated animals will allow us to characterize potential progenitor populations in the POA of the zebrafish.

### Conclusions and future directions

The data reported in this study support a model where there exists a population of hypothalamic progenitor cells that maintain the ability to generate GnRH containing cells throughout life. Our findings are consistent with reports that embryonic rat tissue derived from the striatum subventrical (hypothalamus) has the ability to generate not only neurons and glia, but also GnRH cells (Salvi et al., 2009), that hypothalamic tissue derived from older animals can generate GnRH cells (Markakis et al., 2004), that neurospheres derived from embryonic mice can generate cells containing hypothalamic neuropeptides (Sousa-Ferreira et al., 2011), the loss of hypothalamic GnRH cells in mutants affecting SHH signaling pathways (Whitlock et al., 2003), and the recently proposed role for hypothalamic GnRH in the recuperation of ageing-impaired neurogenesis (Zhang et al., 2013). Additionally our *in vivo* data suggest a neurogenic potential of the hypothalamus similar to that observed in mouse (Bolborea and Dale, 2013; Grandel and Brand, 2013). The ability to generate GnRH cells in the adult animal presents a mechanism that may underlie the reported “reversion” phenotype of hormone therapy in adult males suffering from IHH (Raivio et al., 2007) whereby hormone treatment would induce progenitors to differentiate new GnRH cells, thereby restoring the hormonal environment necessary for reproduction. Because the zebrafish is an excellent model for human disease (Howe et al., 2013) and recent advances in CRISPR/Cas9 technologies will allow us to generate site directed mutations in the zebrafish (Hwang et al., 2013) in genes known to cause Kallmann syndrome our studies present an exciting opportunity to dissect their role in the development and maintenance of GnRH cell progenitors in the central nervous system.

## MATERIALS AND METHODS

### Animals

The New Wild-Type (NWT) and Cornell wild-type lines were generated in the Whitlock laboratory. Both were derived from the wild-type AB genetic background (University of Oregon) by crossing with pet store females and selecting females that produced healthy haploid embryos. The Institutional Animal Use and Care Committee of the Universidad de Valparaíso approved all animal procedures.

### Immunocytochemistry in sectioned tissue

GnRH immunocytochemistry was performed in 1–2 year old male zebrafish that underwent a “mating-training”. This consisted in crossing the animals two times a week for two weeks. Fertile males were selected and sacrificed at 10 AM after being crossed. The brains were fixed in Bouin's solution for 24 h, embedded in paraffin and thin sections (7 µm) were obtained (Cortés-Campos et al., 2011). Alternatively, brains were fixed in PFA 4% with 7% saturated Picric Acid for 4 h at room temperature and thick cryostat sections (20 µm) were obtained (Harden et al., 2012; Whitlock et al., 2003). Immunocytochemistry was carried out as previously described (Cortés-Campos et al., 2011). The primary antibodies used were: mouse anti-GnRH LHR13 (1:100; Park and Wakabayashi, 1986), mouse anti-GnRH HU11B (1:100; Millipore, Temecula, CA, USA; MAB5456), rabbit anti-GnRH BB8 (1:1000; Kah et al., 1986), rabbit anti-mGnRH (1:500; Sigma-Aldrich, St. Louis, MO, USA; G8294) and mouse anti-neurofilament (1:200; Sigma-Aldrich; N2787). Immunoreactivity was visualized using VECTASTAIN^®^ ABC Kit (Vector Laboratories Inc, Burlingame, CA, USA) for thin sections or Alexa-labeled secondary antibodies (1:500; Invitrogen, Rockville, MD, USA) for thick sections. The samples were DNA counter-stained with ferric hematoxilin or DAPI (1:1000; Invitrogen).

### GnRH immunocytochemistry

GnRH is a highly conserved decapeptide with three isoforms described in vertebrates. Most antibodies detect multiple isoforms: Mouse anti-GnRH LHR13 and rabbit anti-sGnRH BB8 detect GnRH3 and the hypothalamic isoform described here, and rabbit anti-mGnRH detects all processed isoforms of GnRH. After initial characterization the BB8 antibody was chosen because it recognizes only hypothalamic GnRH and GnRH3 and, more importantly, allows double labeling with mouse monoclonal neuronal markers.

### Cell culture

Hypothalamic neurospheres cultures were obtained from 1–2 year old animals as previously described (Doetsch et al., 1999) with modifications. Adult fish were sacrificed in ice-cold water and washed in 70% ethanol for 5 min. For each cell culture the hypothalamic area from 8–10 fish were dissected in ice-cold dissection buffer pH 7.4, 320 mOsm/L (20 mM glucose, 44 mM sucrose, 10 mM HEPES, 135 mM NaCl, 5 mM KCl, 0.15 mM Na_2_HPO_4_ and 0.2 mM KH_2_PO_4_). Samples were mechanically disaggregated by up and down pipetting in proliferation medium (Fig. 3A1, isolate progenitors). The medium consisted of NeuroCult^®^ NS-A Basal Medium (STEMCELL Technologies Inc, Vancouver, Canada; 05770) supplemented with NeuroCult^®^ NS-A Proliferation Supplement (STEMCELL Technologies Inc; 05774), 20 ng/ml EGF (STEMCELL Technologies Inc; 02634), 2 mg/ml heparin (STEMCELL Technologies Inc; 07980), 25 ng/ml FGF-b (Sigma-Aldrich; F0291), 100 U/ml penicillin-100 mg/ml streptomycin (Invitrogen), 2.5 mg/ml Fungizone (Invitrogen). The homogenate was decanted over 5 min, the supernatant saved and the pellet re-disaggregated in proliferation medium. Supernatants were mixed and individual cells were seeded at 50–100 cells/ml in culture flasks in proliferation medium (Fig. 3A1, plating progenitors). After 2 days, the cells were centrifuged at room temperature for 5 min at 100 ***g*** and mechanically disaggregated in proliferation medium to obtain individual cells (Fig. 3A2, disaggregation). Cells were cultured in the same medium and cytokines and heparin were added every 2 days. After 5 days the neurospheres (Fig. 3A3, neurospheres) were seeded or dissociated and seeded in NUNC LAB-TEK II CC2 slide 8 chamber coated with 0.2 mg/ml poly-L-lysine (Sigma-Aldrich) and 10 mg/ml laminin (Invitrogen) (Fig. 3A4 upper panel: four plates per culture) and maintained in differentiation medium containing by NeuroCult^®^ NS-A Basal Medium (STEMCELL Technologies Inc, Vancouver, Canada; 05770) supplemented with NeuroCult^®^ NS-A Differentiation Supplement (STEMCELL Technologies Inc; 05773), 100 U/ml penicillin, 100 mg/ml streptomycin, 2.5 mg/ml Fungizone (Invitrogen). The cells were grown up in the same medium for 6 h (Fig. 3A5 upper panel, undifferentiated) or 7 days (Fig. 3A6 upper panel, differentiated) and samples were collected to perform further analyses. The generation of neurospheres from adult zebrafish required rapid and careful dissection in agreement with (Pastrana et al., 2011): a known number of cells were plated, single cell plating was performed, the density was low avoiding the accidental formation of aggregates through mechanical disruption. Quantification was done at consistent times after plating and sphere size was recorded (Fig. 3B-D). All the cells were grown at 28.5°C, 5% CO_2_ in an incubator chamber (Nuaire 5500E).

### Hormone treatment

After one week in proliferation medium, cells were disaggregated in differentiation medium and seeded (see above) (Fig. 3A4, bottom panel). Neurospheres were supplemented every 2 days (3, 5 and 7 day) with vehicle, 10 μM testosterone (Sigma-Aldrich; T1500), or 10 nM GnRH (Sigma-Aldrich; L4897) (prepared in ethanol or PBS respectively, according to manufacturer guidelines). A dose-response curve was performed for GnRH using the following concentrations: 0.01, 0.10, 1.0, 10, 100, 1000 or 10,000 nM. After 5 days of treatment cells were used for immunocytochemistry assays (Fig. 3A5-6, bottom panel).

### Immunocytochemistry of cultured cells

Cells were fixed in PFA 4% for 30 min at room temperature and immunocytochemical procedures were carried out as previously described (Cortés-Campos et al., 2011). The following primary antibodies were used: rabbit anti-sGnRH BB8 (1:1000; Kah et al., 1986), rabbit anti-GFAP (1:200; Dako, Campintene, CA, USA; Z0334), chicken anti-vimentin (1:200; Millipore; AB5733), mouse anti-HUC (1:100; Invitrogen; A-21271), mouse anti-neurofilament (1:200; Sigma-Aldrich; N2787), mouse anti-sox2 (1:1000; Millipore; AB5603), mouse anti-nestin (1:100; BD Biosciences, San Jose, CA, USA; 611659) and mouse anti-PCNA (1:100; Sigma-Aldrich; P8825). The samples were DNA counter-stained with DAPI (1:1000; Invitrogen) and the reactivity revealed using Alexa-labeled secondary antibodies (1:500; Invitrogen).

### Reverse transcription-polymerase chain reaction (RT-PCR)

Total RNA from neurosphere cultures was isolated using Trizol (Invitrogen) and treated with DNase I (Invitrogen). RT-PCR was performed according to the manufacturer's protocol using 1 μg RNA (Invitrogen) and oligodT(20) primer (Invitrogen). The PCR reaction was performed with 1 μl cDNA using the primers previously described to amplify GnRH receptors (Tello et al., 2008) and androgen receptor (Hossain et al., 2008). Each reaction mixture was incubated at 95°C for 5 min followed by 35 cycles of 30 s at 95°C, 30 s at 55°C, and 30 s at 72°C and a final extension of 7 min at 72°C. The expected products were: 582 bp for *GnRHR1*, 765 bp for *GnRHR2*, 421 bp for *GnRHR3*, 298 bp for *GnRHR4*, 237 bp for *AR*, 154 bp for *β-actin* and 192 bp for *gapdh*.

### Microscopy

Bright field images were obtained using a Leica DMR microscope (Leica Microsystems CMS GmbH, Wetzlar, Germany) and a Leica DFC 480 camera (Leica Microsystems Ltd, Heerbrugg, Switzerland); images were processed with the Leica Application Suite 2.3.3 software (Leica Microsystems Ltd). Fluorescent images were taken using a Spinning Disc microscope Olympus BX-DSU (Olympus Corporation, Shinjuku-ku, Tokyo, Japan) and acquired with ORCA IR2 Hamamatsu camera (Hamamatsu Photonics, Higashi-ku, Hamamatsu City, Japan). Images acquired using the Olympus Cell^R software (Olympus Soft Imaging Solutions, Munchen, Germany) were processed using the deconvolution software AutoQuantX 2.2.2 (Media Cybernetics, Bethesda, MD, USA) and ImageJ^®^ software (National Institute of Health, Bethesda, MD, USA).

### Statistical analyses

The total number of cells (DAPI positive), neurons (neurofilament positive) and cells expressing GnRH (BB8 positive) were counted in control and hormone-treated experimental groups and compared by Mann-Whitney-Wilcoxon non-parametric test using GraphPad Prism Version 4.0 software (GraphPad Software, San Diego, CA, USA).

## Supplementary Material

Supplementary information
