# Supplementary material for: Zebrafish adult-derived hypothalamic neurospheres generate gonadotropin-releasing hormone (GnRH) neurons
Source: Biol Open. 2015 Jul 24;4(9):1077–86. doi: 10.1242/bio.010447 (PMC4582115; doi:10.1242/bio.010447)
Supplement: Supplementary information [file supp_4_9_1077__index.html]

Zebrafish adult-derived hypothalamic neurospheres generate gonadotropin-releasing hormone (GnRH) neurons — Supplementary information 

# Zebrafish adult-derived hypothalamic neurospheres generate gonadotropin-releasing hormone (GnRH) neurons

## BIO010447 Supplementary information

**Files in this Data Supplement:**

- Supplementary information
