# Supplementary material for: Zebrafish adult-derived hypothalamic neurospheres generate gonadotropin-releasing hormone (GnRH) neurons
Source: Biol Open. 2015 Jul 24;4(9):1077–86. doi: 10.1242/bio.010447 (PMC4582115; doi:10.1242/bio.010447)
Supplement: Supplementary information [file supp_bio.010447_BIO010447supp.pdf]

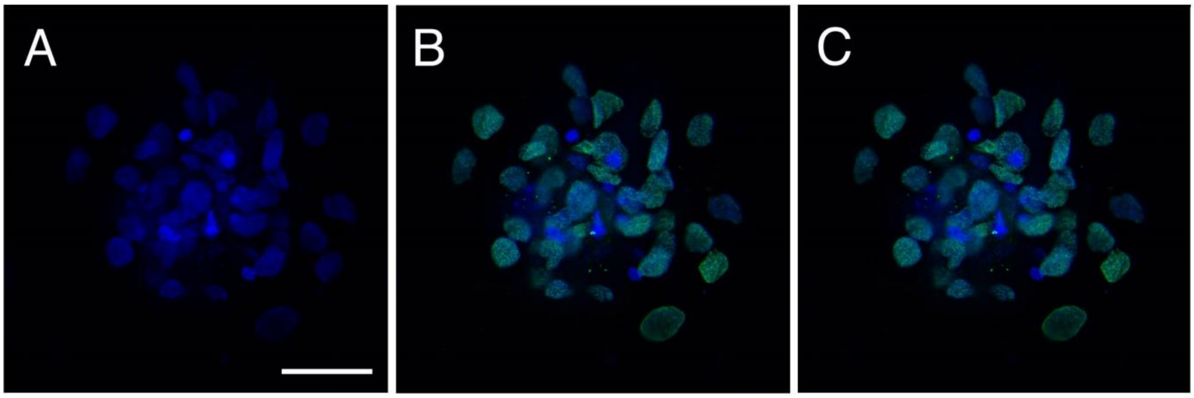

Fig. S1. Undifferentiated secondary neurospheres do not express GnRH. Undifferentiated secondary neurospheres were labeled with DAPI, anti-GnRH antibody (LHR13; A, no expression detected), anti-Sox2 antibody (B, green), with all channels shown in C. All Images: anterior is to the left. Scale bar=30µm.
